# Identification and characterization of a novel heparinase PCHepII from marine bacterium *Puteibacter caeruleilacunae*

**DOI:** 10.1038/s41598-023-47493-y

**Published:** 2023-11-17

**Authors:** Danrong Lu, Luping Wang, Zeting Ning, Zuhui Li, Meihua Li, Yan Jia, Qingdong Zhang

**Affiliations:** School of Life Science and Technology, Weifang Medical University, 7166 Baotong West Street, Weifang, 261053 China

**Keywords:** Carbohydrates, Enzyme mechanisms, Glycobiology

## Abstract

Heparin (HP) and heparan sulfate (HS) are multifunctional polysaccharides widely used in clinical therapy. Heparinases (Hepases) are enzymes that specifically catalyse HP and HS degradation, and they are valuable tools for studying the structure and function of these polysaccharides and for preparing low molecular weight heparins. In this study, by searching the NCBI database, a novel enzyme named PCHepII was discovered in the genome of the marine bacterium *Puteibacter caeruleilacuae*. Heterologously expressed PCHepII in *Escherichia coli* (BL21) has high expression levels and good solubility, active in sodium phosphate buffer (pH 7.0) at 20°C. PCHepII exhibits an enzyme activity of 254 mU/mg towards HP and shows weak degradation capacity for HS. More importantly, PCHepII prefers to catalyse the high-sulfated regions of HP and HS rather than the low-sulfated regions. Although PCHepII functions primarily as an endolytic Hepase, it mainly generates disaccharide products during the degradation of HP substrates over time. Investigations reveal that PCHepII exhibits a preference for catalysing the degradation of small substrates, especially HP tetrasaccharides. The catalytic sites of PCHepII include the residues His^199^, Tyr^254^, and His^403^, which play crucial roles in the catalytic process. The study and characterization of PCHepII can potentially benefit research and applications involving HP/HS, making it a promising enzyme.

## Introduction

Glycosaminoglycans are a group of polyanionic and heterogeneous polysaccharides containing heparin (HP) and heparan sulfate (HS). HP and HS consist of repeating disaccharides, which consist of D-glucuronic acid/L-iduronate acid (GlcA/IdoA) and *N*-acetyl-D-glucosamine (GlcNAc)^1^. These polysaccharides have a broad distribution, and they are present on the surfaces of cells, in the extracellular matrix (ECM), and even within the intracellular environment (found in mast cells)^2^. Modification of the common precursor of HP and HS involves multiple enzymes, such as sulfotransferases that can selectively add sulfate groups to GlcA/IdoA (2S) or GlcNAc (3S, 6S, NS) residues and epimerases that can convert some GlcA to IdoA, resulting in highly complex HP and HS^3,4^. As a result, the HP polysaccharides are mainly composed of the trisulfated disaccharide unit -4 IdoA2S 1–4 GlcNS6S, and the degree of sulfation was approximately 2.7. HS mainly consists of lower-sulfated disaccharide units, and the degree of sulfation is lower than that of HP^5^. HP and HS, which are composed of numerous variations of disaccharides, are the most intricate polymers that exist in nature^4^.

The intricacy of the HP and HS structure allows them to participate in numerous physiological and pathological mechanisms, for example, HP is known to function as an anticoagulant^6^. Since its identification in the 1920s, HP and low molecular weight heparins (LMWHs) have been extensively utilized as a prominent category of anticoagulants in clinical therapy^1^. In addition, HP and its related compounds have been found to exhibit various other biological functions, including roles in cellular adhesion^7^, inflammation^8^, cellular migration^9^, differentiation^10^, and even pathogenic infection^11^. These functions are mediated through interactions with different signalling proteins^7,12,13^. Given the significant biological functions of HP and its related compounds, there has been considerable interest in both structural and functional research on these compounds, as well as their clinical applications.

**Figure 1:**
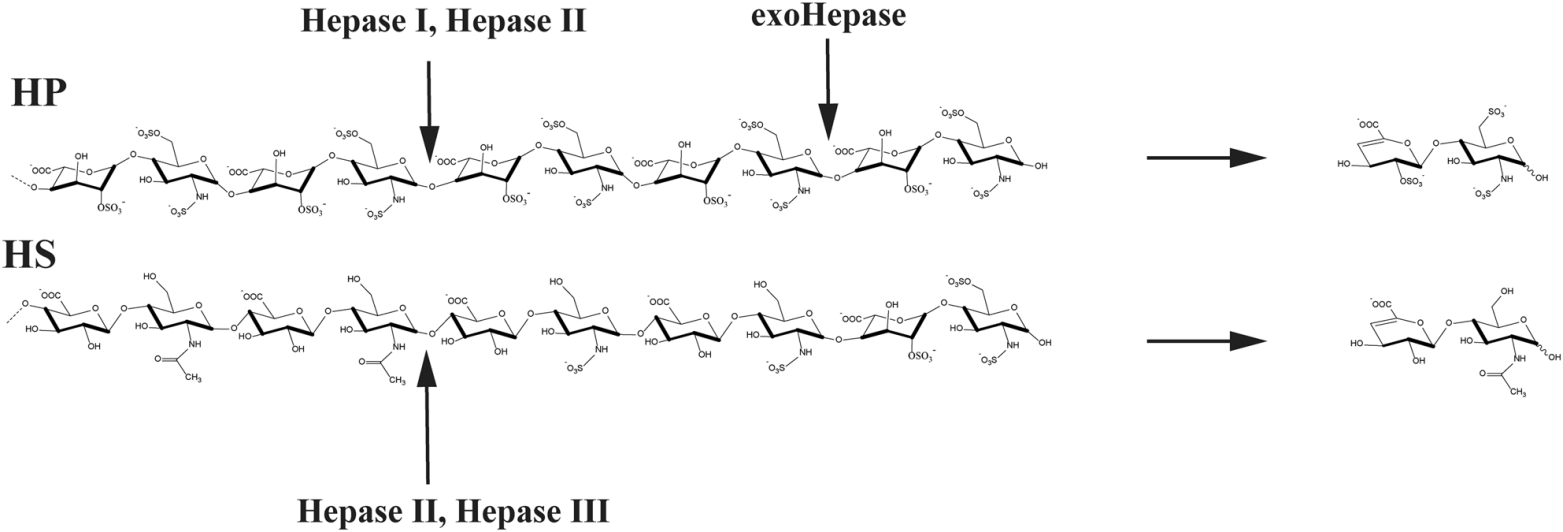
Substrate preference of Hepase I, Hepase II and Hepase III.

Heparinases (Hepases) are a group of enzymes in the polysaccharide lyase families derived from bacteria. They utilize a β-elimination reaction to breakdown HP and HS and produce oligosaccharide products with unsaturated double bonds at the nonreducing end^12^. Hepases can be classified into three types: Hepase I (EC4.2.2.7), Hepase II (EC 4.2.2.-), and Hepase III (EC 4.2.2.8), each with distinct substrate preferences: Hepase I prefers to catalyse high-sulfated HP, Hepase III prefers to degrade low-sulfated HS, and Hepase II can effectively digest both polysaccharides (Fig. 1)^14^. The widely used Hepases are Hepase I, Hepase II, and Hepase III from *Pedobacter heparinus* DSM 2366 (formerly known as *Flavobacterium heparinum*)^15–17^. These Hepases are typically employed to examine the precise composition of HP and HS and for the preparation of LMWHs. For example, the widely used drug tinzaparin is prepared via the partial degradation of HP polysaccharides by Hepase I^18^.

The commercial hepases currently available are mainly extracted from fermentation broths of *P. heparinus* DSM 2366, but they have a relatively high selling price. Therefore, researchers have turned to heterologous hepases from *P. heparinus* DSM 2366 and to the identification of novel hepases from other bacteria. New variants of Hepase I, Hepase II and Hepase III have been discovered in various bacterial strains, such as *Bacteroides stercoris* HJ-15^19–21^, *Bacteroides thetaiotaomicron* (BT4657)^14,22^, *Bacillus circulans* HpT298^23^, *Prevotella heparinolytica*^24^, *Bacteroides eggerthii* VPI T5-42B-1^25^, *Bacteroides cellulosilyticus*^26^, *Raoultella* sp. NX-TZ-3-15^27,28^, and *Streptomyces variabilis* MTCC 12266^29^. Additionally, new families of Hepases, known as exolytic Hepases, have recently been identified^30^. However, while Hepases derived from terrestrial bacteria have been extensively researched and reported, the novel Hepases from specialized marine environments that may possess novel characteristics have not yet been documented^31^.

This research presents the identification and report of the novel Hepase PCHepII, an enzyme from a marine bacterium. This study provides a detailed analysis of the enzyme's properties, including its enzymatic characteristics, substrate catalytic modes, substrate preferences, and structure of the catalytic sites. The marine hepase PCHepII exhibits efficient degradation capabilities, suitable enzymatic properties, and a preference for catalysing small-sized oligosaccharide substrates. The results indicate that PCHepII may serve as a beneficial tool to perform structural investigations on HP and HS.

## Material and methods

### Materials

The PCHepII gene from *Puteibacter caeruleilacunae* (GenBank code: TKG96895.1) was selected from the National Center for Biotechnology Information (NCBI) GenBank database. Vazyme Biotech, Inc. provided the *Escherichia coli* competent BL21 cells (DE3) (Nanjing, China). Hyaluronate (HA) (BR degree), chondroitin sulfate (CS) (BR degree), dermatan sulfate (DS) (BR degree), sodium alginate (BR degree), 2-aminobenzamide (2-AB), and sodium cyanoborohydride (NaBH_3_CN) were obtained from Sigma‒Aldrich Inc. Hepase I, Hepase II and Hepase III were provided by the Fuchuan Li Laboratory. HP (BR degree) from porcine intestinal mucosa was purchased from Hefei BOMEI Biotechnology Co., Ltd. (Anhui, China). HS was extracted from porcine intestinal mucosa^32^. The HP oligosaccharides were prepared by partially digesting HP polysaccharides using Hepase I followed by gel filtration chromatography on a Superdex 30 Increase 10/300 GL column, and the detailed methods are provided in the Supplementary materials. China National Medicines Corporation Ltd. (Beijing, China) supplied all the chemicals and reagents mentioned in this paper.

### Sequence analysis of the PCHepII gene and protein

The DNA and protein sequences of PCHepII were obtained from the NCBI database. The G + C% content of pchepII was computed using Bio-Edit version 7.0.9. The theoretical molecular weight (Mw) and isoelectric point (pI) of the PCHepII protein were determined using the ExPASy server of the Swiss Institute of Bioinformatics and the peptide estimation tool. To determine the type of PCHepII and its corresponding secretion signal peptide, the SignalP 5.0 server was used for prediction. Phylogenetic analysis and amino acid alignment, including that of newly identified hepases, were performed using MEGA version 7.0. To gain insight into the function and sequence similarity of the enzymes, functional annotation and sequence similarity analysis were carried out by searching for amino acid sequences online using the NCBI's online BLAST algorithm. Identification of the protein modules and functional domains of PCHepII was achieved by utilizing SMART, the Pfam database, and the Carbohydrate-Active Enzyme database.

### Heterologous expression and purification of PCHepII

The *pchepII* gene was artificially synthesized by the company GENEWIZ, Inc., located in Suzhou, China. Then, it was combined with the vector pET-30a ( +) to generate the recombinant plasmid pET30a-*pchepII*, which carries a C-terminal (His)_6_-tag. The recombinant plasmid was transformed into competent *E. coli* BL21 (DE3) cells by heat shock at 42°C for 1 min. Positive colonies of *E. coli* BL21-pET30a-*pchepII* were verified by the gene sequencing process in Sangon Biotech (Shanghai, China) and cultivated in fresh LB broth supplemented with kanamycin at a final concentration of 0.05 mg/mL. Cultivation was carried out at 37°C and 200 rpm until the cell density reached 0.6–0.8 (OD_600_). Once the desired cell density was achieved, the mixture was cooled to 16°C, and agitation at 220 rpm was employed to induce the synthesis of the target protein PCHepII. This was achieved by adding 0.05 mM IPTG and allowing protein expression to proceed for 18–24 h.

For the subsequent steps, the fermentation broth was resuspended in prechilled buffer A, which consisted of 50 mM Tris–HCl and 150 mM NaCl and had a pH of 8.0. Centrifugation was then carried out at 8000 × *g* for 10 min to collect the cells. Next, the cells were subjected to ultrasonication (72 cycles, 4 s each) to rupture them. The resultant mixture was subjected to centrifugation at 15,000 × *g* for 30 min. This supernatant was collected and introduced onto a column packed with nickel-Sepharose™ 6 Fast Flow (manufactured by GE Healthcare). Prior to sample loading, the column was equilibrated with buffer A. Elution of the target protein PCHepII was accomplished by using buffer A containing a linear concentration of imidazole that ranged from 0 to 500 mM, and then the 250 mM eluate was collected. The protein samples were reduced and then assessed by performing SDS‒PAGE using a 12% (w/v) gel to ensure the purity and molecular weight of purified PCHepII. ImageJ software was applied to calculate the purity of PCHepII. To ensure removal of impurities, overnight dialysis at 4°C was conducted. The protein concentration of the purified PCHepII was quantified by employing a bicinchoninic acid (BCA) protein assay kit manufactured by Sangon Biotech in Shanghai, China.

### Substrate specificity analysis of recombinant PCHepII

To explore the substrate specificity of PCHepII, a variety of polysaccharides were employed as substrates, including HA, CS, DS, HP, HS, and alginate. To completely degrade the polysaccharide substrates (1 mg/mL), 6 μg of the enzyme was introduced into 50 mM sodium phosphate buffer (NaH_2_PO_4_-Na_2_HPO_4_ buffer) (pH 8.0) and incubated at 30°C for 24 h. To establish a negative control, the same amount of inactivated enzyme was applied to the negative control group. Following the digestion reaction, the system was subjected to several procedures: boiling for 10 min to halt the reaction, cooling in an ice-water bath for 5 min, and centrifugation at 12,000 rpm for 10 min to eliminate insoluble matter. Subsequently, the acquired supernatants were analysed for unsaturated products based on solution absorbance at 232 nm or gel filtration high-performance liquid chromatography (HPLC).

### Biochemical characterization investigation of PCHepII

The impact of temperature on PCHepII enzyme activity was investigated by establishing reaction systems with HP as a substrate in 50 mM sodium phosphate buffer (pH 8.0) at temperatures ranging from 0 to 70°C for a duration of 30 min. To determine the optimal reaction buffer and pH, various reaction buffers, including NaAc-HAc buffer (50 mM, pH 5.0–6.0), sodium phosphate buffer (50 mM, pH 6.0–8.0), and Tris–HCl buffer (50 mM, pH 7.0–10.0), were employed. To determine the thermostability of PCHepII, the enzyme was incubated at varying temperatures (0–50°C) for 0 h to 24 h, and residual enzyme activity on HP was measured at the optimal temperature and pH. The effects of metal ions, chelators and LiCl on the HP-degrading activity of PCHepII were determined via supplementation with various metal ions/chelators at a concentration of 5 mM and different concentrations of LiCl or NaCl ranging from 0–500 mM. The metal ions and chelators included Li^+^, K^+^, Na^+^, Ag^+^, Ca^2+^, Mg^2+^, Mn^2+^, Ni^2+^, Co^2+^, Hg^2+^, Cd^2+^, Zn^2+^, Pb^2+^, Fe^2+^, Ba^2+^, Cu^2+^, Fe^3+^, Cr^3+^, EDTA, imidazole, glycerol, SDS, β-mercaptoethanol and DTT. All assays were carried out in triplicate. The PCHepII activity value was determined by averaging the absorbance at 232 nm for parallel reaction solutions.

To obtain the enzyme activity of PCHepII on HP and HS, reaction systems containing 30 μL of enzyme (1 μg/μL), 30 μL of HP or HS (10 mg/mL), 10 μL of LiCl (3 M), 10 μL of BaCl_2_ (150 mM), 100 μL of sodium phosphate buffer (150 mM, pH 7.0) and 150 μL of deionized water were employed. Incubation of the samples was carried out at 20°C for 0–5 min, after which boiling water was applied for 10 min to halt the digestion reaction. Determination of PCHepII activity involved measuring the absorbance change per min at 232 nm, with an extinction coefficient of 3800 M^−1^ cm^−1^ for degradation products [1 U = 1 μmol of unsaturated carbon bond-containing product formed per min].

To test the kinetic parameters (*K*_m_ and *V*_max_) of PCHepII, the enzyme activities using different concentration of HP at the optimal reaction condition were measured. Kinetic parameters were derived from Michaelis–Menten representations.

### Anion exchange HPLC analysis of the reaction system with PCHepII and PHHepII

Anion exchange HPLC was applied to analyse the final products of HP and HS degradation by PCHepII. Each 30 μg of HP and HS was treated with 1.2 mU PCHepII or PHHepII at 20°C or 37°C for 24 h. The reaction mixtures were boiled for 10 min and centrifuged at 15,000 × *g* for 10 min. Then, the samples were filtered through 0.22 μm filters followed by analysis using anion exchange HPLC on a Pack Polyamine II column (YMC). The elution process was performed using a linear NaH_2_PO_4_ gradient of 0.016–1.0 M at a flow rate of 1.0 mL/min over a 60 min period, and the fractions were monitored at 232 nm using a UV detector. Standard unsaturated disaccharides were coinjected, and their retention times were compared to identify reaction products.

### Polysaccharide degradation mode analysis of PCHepII

To investigate the PCHepII degradation mode of polysaccharides, a reaction system containing 100 μL of enzyme (0.2 μg/μL), 100 μL of HP (10 mg/mL), 100 μL of LiCl (1 M), 33 μL of BaCl_2_ (150 mM), 333 μL of sodium phosphate buffer (150 mM, pH 7.0) and 334 μL of deionized water was employed. The mixture was then incubated at 20°C for 0 min, 10 min, 30 min, 1 h, 2 h, and 24 h. During this incubation period, 60 μL samples of the reaction mixture were extracted and boiled for 10 min. The samples were centrifuged at 15,000 × *g* for 10 min and then filtered through 0.22 μm filters followed by analysis using gel filtration HPLC on a Superdex 30 Increase 10/300 GL column (Cytiva). The elution process was performed using 0.2 M NH_4_HCO_3_ at a flow rate of 0.4 mL/min, and the fractions were monitored at 232 nm using a UV detector.

### Effects of fluorescence labelling on the digestion of oligosaccharides by PCHepII

To examine the influence of fluorescence labelling on the degradation of oligosaccharides by PCHepII, unsaturated HP oligosaccharides tetrasaccharide (UDP4) and hexasaccharide (UDP6) were subjected to 2-AB labelling^33^. Next, 0.5 μg of the labelled samples were treated with 1 mU of PCHepII under the optimal conditions. The samples were subjected to a 10 min boiling procedure and were subsequently filtered using 0.22 μm filters. The resulting supernatants were assessed with gel filtration HPLC (Thermo Fisher UltiMate 3000) on a Superdex 30 Increase 10/300 GL column (Cytiva). The elution was carried out with 0.2 M NH_4_HCO_3_ at a flow rate of 0.4 mL/min. To monitor the process, a fluorescence detector with excitation and emission wavelengths of 330 and 420 nm, respectively, was utilized.

### Substrate size preference analysis of PCHepII

To investigate the substrate size preference of PCHepII, a variety of unsaturated HP oligosaccharides with different size types were applied. Specifically, 2 nmol of each of the unsaturated HP oligosaccharides (UDP4, UDP6, UDP8, and UDP10) was treated with 0.16 μU PCHepII at the optimal conditions for various time intervals: 0 min, 1 min, 5 min, 10 min, and 30 min. The samples were extracted from the reaction system, boiled for 10 min and then filtered using 0.22 μm filters. Subsequently, the samples were analysed via UV gel-filtration HPLC as described in Sect. “Polysaccharide degradation mode analysis of PCHepII”.

### Site-directed mutagenesis of PCHepII

To determine the active centre of PCHepII, the 3D structure of PCHepII was modelled using Swiss Model online software (https://swissmodel.ExPASy.org/), with the Hepase II structure (PDB code: 2FUT) as the template. The candidate residues His^199^, Tyr^254^, and His^403^ were selected for further analysis according to alignment with the active sites of PHHepII and mutated to Ala using the Mut Express II Fast Mutagenesis Kit V2 (Vazyme, Nanjing, China). The primers included H199A-F: TAGCATTACGAGCGCGACGAGCGAATGGATGCTGAT, H199A-R: TCGCGCTCGTAATGCTATACTGTTTAATCGGC, Y254A-F: AACAGCGCGTACAACGTGCGCTTTAGCAGCGA, Y254A-R: ACGTTGTACGCGCTGTTGCCTTGATGATAGGTATG, and H403A-F: TTCTGAACGCGCAGCATCATGACGCGGGCGCG. The residual enzyme activities of the mutants PCHepII-H199A, PCHepII-Y254A, and PCHepII-H403A towards HP and HS were analysed as described in 2.4 and 2.5.

## Results

### Sequence features of the PCHepII gene and protein

**Figure 2:**
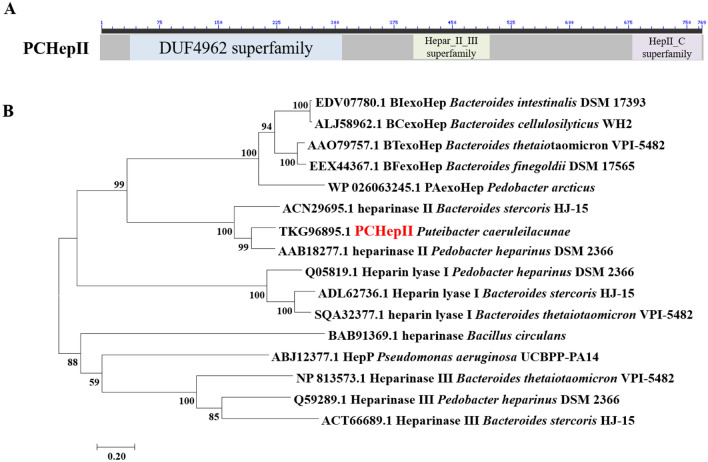
Module organization (**A**) and phylogenic analysis (**B**) of PCHepII. MEGA was used to conduct a phylogenetic analysis with the neighbour-joining method, resulting in taxa clustering together during the 1000 bootstrap test.

The length of the gene that encodes PCHepII is 2310 bp, and its GC content is 40.78%. PCHepII is a protein that consists of 769 amino acids. At the N-terminus, it contains a signal peptide consisting of 23 amino acids. The molecular mass of the PCHepII protein was calculated to be 88.26 kDa, and it has an isoelectric point value (pI) of 7.00. Analysis of the sequence revealed that PCHepII possesses three conserved domains. These domains include a DUF4962 superfamily domain at the N-terminus (Pro^42^-Pro^312^), a Hepar_II_III superfamily domain on the interior (His^403^-Pro^312^), and a HepII_C domain at the C-terminus (Ile^684^-Arg^769^) (Fig. 2A). Phylogenetic analysis showed that PCHepII was closely related to other identified Hepases II (Fig. 2B), particularly the Hepase II (PHHepII) from *P. heparinus* DSM 2366, a commercial Hepase II. Further similarity analysis revealed that PCHepII shares the highest amino acid identity of 68.07% with PHHepII (GenBank code: AAB18277.1)^14^. These results suggested that PCHepII can be inferred to be a novel Hepase and may function similarly to Hepase II, which degrades HP and HS.

### Heterologous expression and purification of recombinant PCHepII

**Figure 3:**
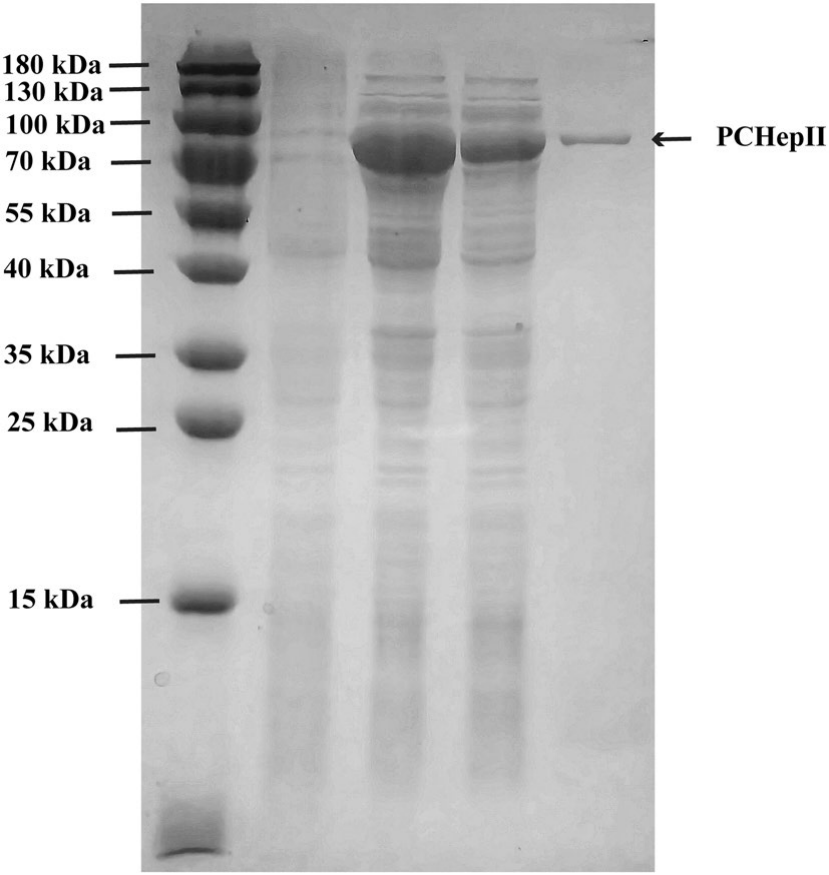
SDS-PAGE analysis of recombinant PCHepII expressed in *E. coli*. Coomassie brilliant blue was used to stain 12% (w/v) SDS-PAGE gels for the analysis of the crude extract and purified PCHepII. Lane 1, pre-stained protein standard marker (15–180 kDa); Lane 2, uninduced cell lysate of *E. coli*-pET30a; Lane 3, IPTG-induced cell lysate of *E. coli*-pET30a-*pchepII*; Lane 4, the induced lysate supernatant of *E. coli*-pET30a-*pchepII*; Lane 5, purified PCHepII after Ni^2+^ affinity chromatography.

The recombinant plasmid pET30a-*pchepII* was synthesized by GENEWIZ, Inc. (Suzhou, China) and successfully overexpressed the soluble protein without the signal peptide in *E. coli* BL21 (DE3) at 16°C. As shown in Fig. 3, the intracellular target protein PCHepII was released into the supernatant after the cells were subjected to ultrasonication and centrifugation. The soluble fractions were then purified using a Ni^2+^ affinity column based on the (His)_6_-tag. The SDS‒PAGE results indicated that the purification procedure was highly effective, resulting in a single band, and the purity of purified PCHepII was calculated to be 100%. Based on a comparison with the prestained protein marker, the molecular weight of the single band was estimated to be approximately 90 kDa, which is consistent with the theoretical molecular weight (88.26 kDa) of PCHepII.

### Biochemical characterization of recombinant PCHepII

Substrate specificity experiments demonstrated that PCHepII displayed a notable variation in the degradation of different substrates. Specifically, PCHepII effectively degraded HP (Supplementary Fig. 1A), exhibited weak degradation of HS (Supplementary Fig. 1B), and showed no activity towards substrates such as HA, CS, DS, and alginate (Supplementary Fig. 1C–F). These findings indicate that the recombinant PCHepII functioned as a Hepase that primarily catalyses the high-sulfated substrate HP, rather than the low-sulfated substrate HS.

**Figure 4:**
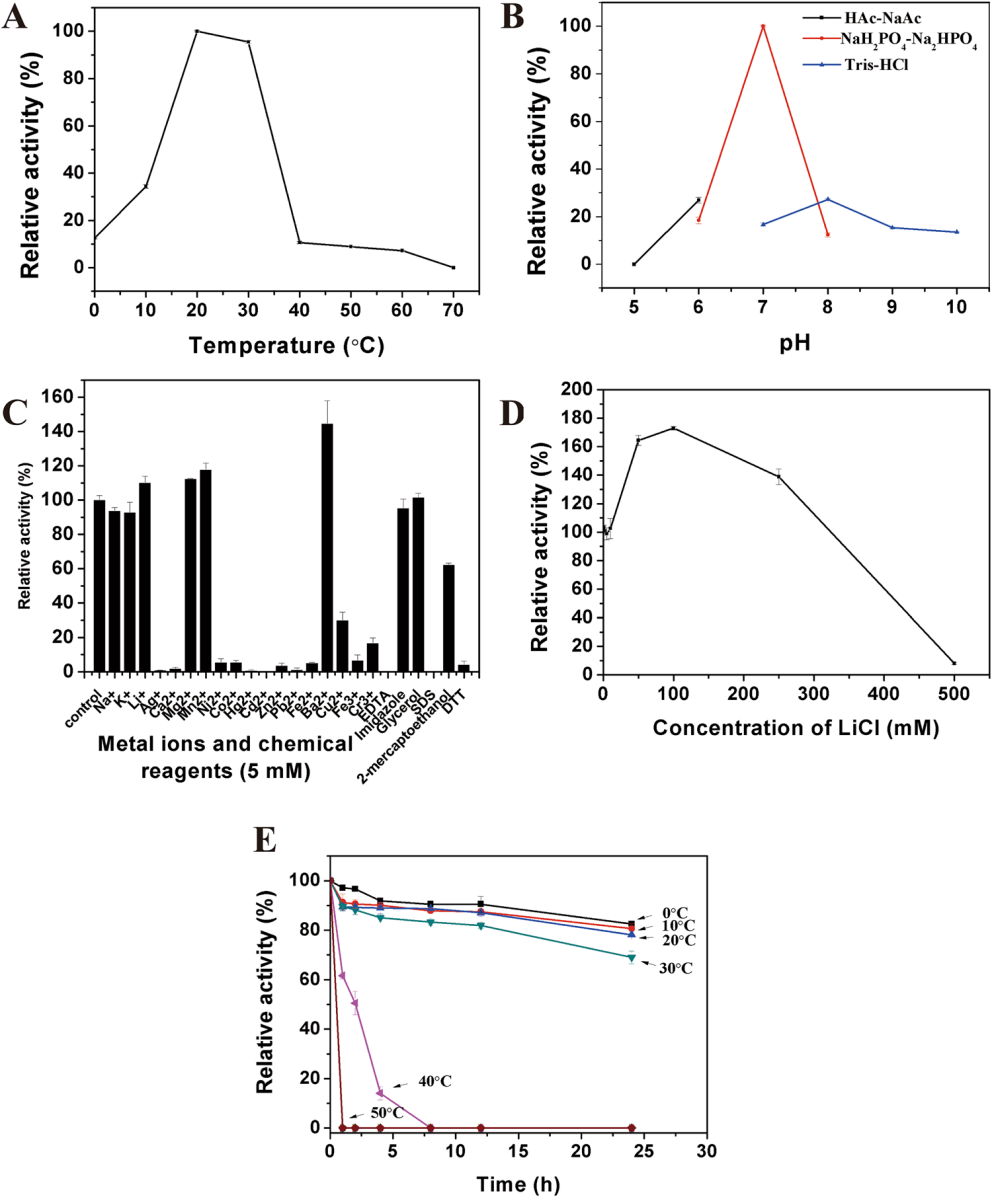
Biochemical characterization of recombinant PCHepII. (**A**) Effect of temperature on PCHepII activity. The highest activity observed at 20°C on HP was set at 100%. (**B**) Effect of pH on PCHepII activity. The highest activity observed in the 50 mM sodium phosphate buffer (pH 7.0) on HP was set as 100%. (**C**) Effect of metal ions and chelators including Li^+^, K^+^, Na^+^, Ag^+^, Ca^2+^, Mg^2+^, Mn^2+^, Ni^2+^, Co^2+^, Hg^2+^, Cd^2+^, Zn^2+^, Pb^2+^, Fe^2+^, Ba^2+^, Cu^2+^, Fe^3+^, Cr^3+^, EDTA, imidazole, glycerol, SDS, β-mercaptoethanol and DTT on PCHepII activity. The enzyme activity on HP obtained without the inclusion of tested metal ions and chemicals was considered as 100%. (**D**) Effect of KCl concentration on PCHepII activity. The activity of PCHepII without LiCl was set as 100%. (**E**) Thermostability of PCHepII. The residual activity of PCHepII on HP without preincubation was set at 100%.

Biochemical characterization experiments were conducted to optimize the enzyme activity of PCHepII. The impact of temperature on PCHepII enzyme activity was tested over a temperature range of 0°C to 70°C. The maximum activity based on the production of unsaturated degradation products was observed at 20°C, and the activity sharply declined when the temperature was over 30°C (Fig. 4A). To assess the thermostability of PCHepII, its enzyme activity was examined under various temperature conditions ranging from 0°C to 50°C. The results showed that PCHepII was relatively stable at 0–30°C (Fig. 4E). The stability of PCHepII sharply declined when the temperature was higher than 30°C. To test the influence of different reaction buffers and pH values on the HP-degrading activity of PCHepII, NaAc-HAc buffer (50 mM, pH 5.0–6.0), sodium phosphate buffer (50 mM, pH 6.0–8.0), and Tris–HCl buffer (50 mM, pH 7.0–10.0) were employed. The results revealed that the highest HP-degrading activity occurred at pH 7.0 in the sodium phosphate buffer (Fig. 4B), and other pH values may have a negative impact on the enzyme activity of PCHepII.

Moreover, the impact of various chemicals and metal ions (5 mM), including Li^+^, K^+^, Na^+^, Ag^+^, Ca^2+^, Mg^2+^, Mn^2+^, Ni^2+^, Co^2+^, Hg^2+^, Cd^2+^, Zn^2+^, Pb^2+^, Fe^2+^, Ba^2+^, Cu^2+^, Fe^3+^, Cr^3+^, EDTA, imidazole, glycerol, SDS, β-mercaptoethanol and DTT, on the enzyme activity of PCHepII was investigated under optimum conditions (Fig. 4C). The presence of specific divalent cations, including Mg^2+^, Mn^2+^, and Ba^2+^, substantially enhanced the HP-degrading activity of PCHepII. Among these cations, Ba^2+^ exhibited the most pronounced positive effect, resulting in a 145% increase in activity (Fig. 4C). Conversely, the addition of EDTA completely inhibited the enzyme activity of PCHepII, implying the essential role of divalent cations in its catalytic function. On the other hand, chemicals and metal ions, including Na^+^, K^+^, imidazole, and glycerol, did not show an obvious influence on PCHepII enzyme activity; β-mercaptoethanol slightly inhibited PCHepII enzyme activity; Ni^2+^, Co^2+^, Zn^2+^, Cu^2+^, Fe^2+^, Fe^3+^, Cr^3+^, and DTT severely inhibited the HP-degrading activity of PCHepII; and Ca^2+^, Ag^+^, Pb^2+^, Hg^2+^, Cd^2+^, and SDS completely inhibited the HP-degrading activity of PCHepII. Additionally, the presence of LiCl greatly stimulated the HP-degrading activity of PCHepII, with the most significant enhancement (173%) observed at a concentration of 100 mM (Fig. 4D). Even at a concentration of 250 mM, enzyme activity was enhanced (Fig. 4D). However, the presence of NaCl in the reaction system did not significantly stimulate the activity of PCHepII, the use of 100 mM NaCl resulted in a 113% increase in enzyme activity (Supplementary Fig. 2).

The specific activity and kinetic parameters of PCHepII towards HP were measured by calculating the absorbance change per min at 232 nm. As a result, the enzyme activity of PCHepII towards HP was measured to be 254 mU/mg protein, and the *V*_max_ and *K*_m_ of PCHepII were calculated as 494 mU/mg protein and 0.33 mg/mL, respectively. While, the enzyme activity towards HS was too low to measure, although PCHepII could partially degrade HS (Supplementary Fig. 1B). These results suggest that the novel PCHepII exhibits a stronger preference for catalysing high-sulfated HP rather than low-sulfated HS.

### Anion exchange HPLC analysis of the reaction system with PCHepII and PHHepII

**Figure 5:**
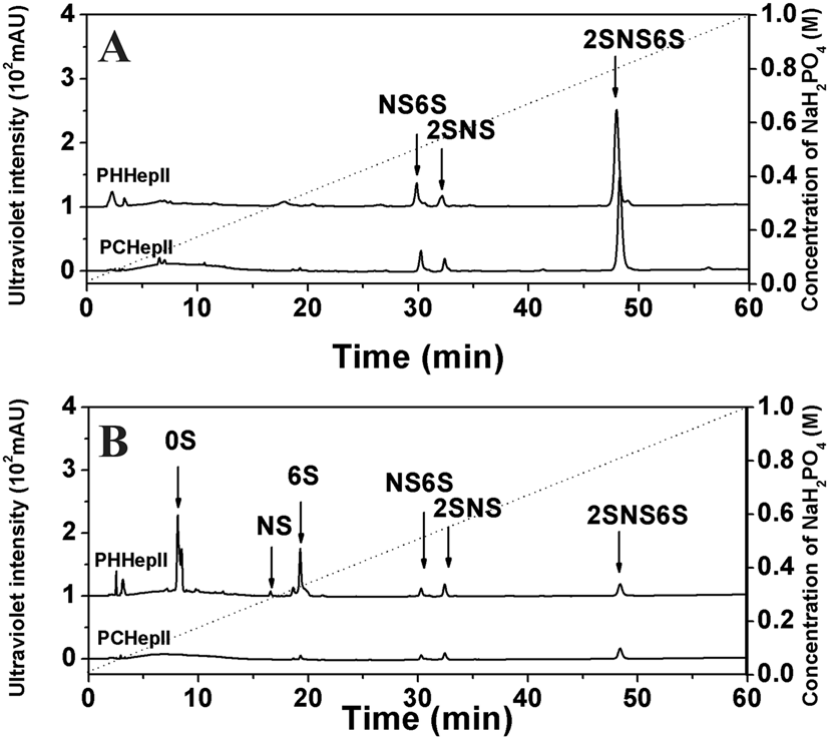
Final products of HP and HS degraded by PCHepII and PHHepII. The final products of HP (**A**) and HS (**B**) by PCHepII and PHHepII were analyzed via the anion exchange HPLC. 0S, ΔUA(1–4)GlcNAc; 6S, ΔUA(1–4)GlcNAc6S; 2S, ΔUA2S(1–4)GlcNAc; NS, ΔUA(1–4)GlcNS; 2S6S, ΔUA2S(1–4)GlcNAc6S; NS6S, ΔUA(1–4)GlcNS6S; 2SNS, ΔUA2S(1–4)GlcNS; 2SNS6S, ΔUA2S(1–4)GlcNS6S.

The final products of HP and HS degradation by PCHepII and PHHepII were analysed via anion exchange HPLC. The results showed that the final products of HP degradation by PCHepII and PHHepII were similar and were mainly composed of the trisulfated disaccharide ΔUA2S1-4GlcNS6S and a small quantity of the disulfated disaccharides ΔUA2S1-4GlcNS and ΔUA1-4GlcNS6S (Fig. 5A). The final products of HS degradation by PCHepII were composed of the trisulfated disaccharide ΔUA2S1-4GlcNS6S and the disulfated disaccharides ΔUA2S1-4GlcNS and ΔUA1-4GlcNS6S (Fig. 5B). The final products of HS degradation by PHHepII were composed of the trisulfated disaccharide ΔUA2S1-4GlcNS6S, the disulfated disaccharides ΔUA2S1-4GlcNS and ΔUA1-4GlcNS6S, the monosulfated disaccharides ΔUA1-4GlcNS and ΔUA1-4GlcNAc6S, and the nonsulfated disaccharide ΔUA1-4GlcNAc (Fig. 5B). The results indicated that PCHepII preferred to degrade the high-sulfated regions of HP and HS, while PHHepII could degrade both the high-sulfated and low-sulfated regions of HP and HS.

### Polysaccharide degradation mode of PCHepII

**Figure 6:**
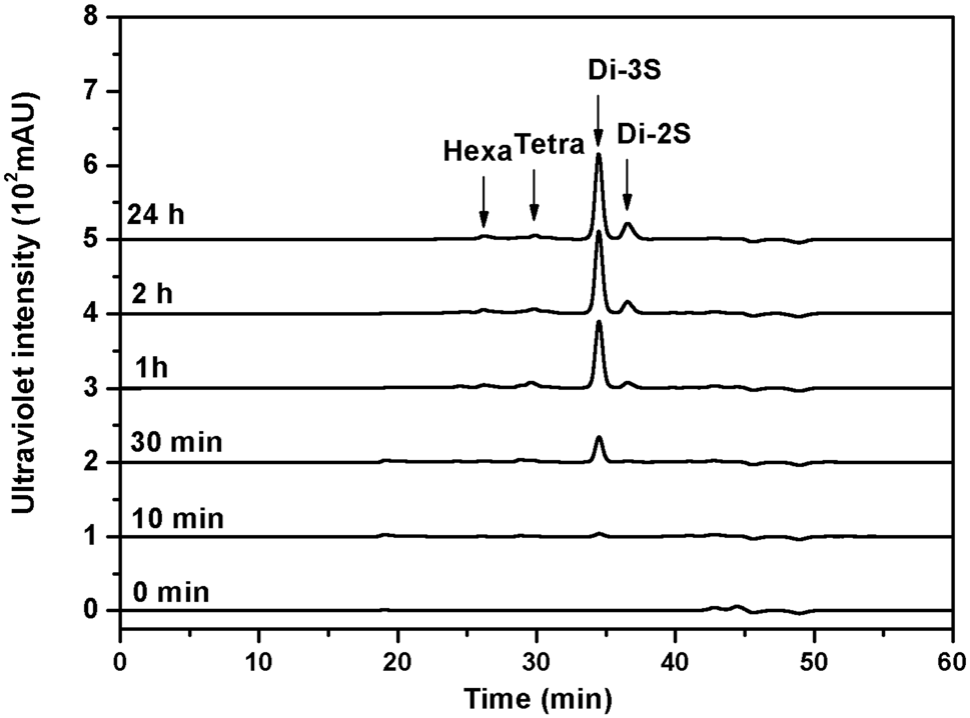
Polysaccharide degradation pattern analysis of recombinant PCHepII with HP as substrate. Purified PCHepII was used to treat HP (1 mg/mL) at 20°C in 50 mM sodium phosphate buffer (pH 7.0) for 0–24 h. Samples of 30 μg were taken at intervals for gel filtration HPLC analysis. The degree of depolymerization of HP unsaturated oligosaccharides released from the polysaccharide substrates by the digestion of HP are indicated by arrows: Hexa, HP unsaturated hexasaccharide; Tetra, HP unsaturated tetrasaccharide; Di-3S, the trisulfated HP unsaturated disaccharides; Di-2S, the disulfated HP unsaturated disaccharides.

The mechanism of PCHepII action was studied via the degradation of HP under optimal conditions for 0–24 h. Reaction solutions for quantification were collected at regular time intervals and analysed using HPLC at 232 nm. Initially, the primary degradation (30 min as an example) products of PCHepII were unsaturated oligosaccharides with high molecular weights, as shown in Supplementary Fig. 3. As the reaction time increased, the molecular size of these unsaturated oligosaccharides gradually decreased, eventually forming unsaturated disaccharides. These findings indicate that PCHepII is an endo-type Hepase, as shown in Fig. 6.

### Influence of fluorescence labelling on PCHepII

**Figure 7:**
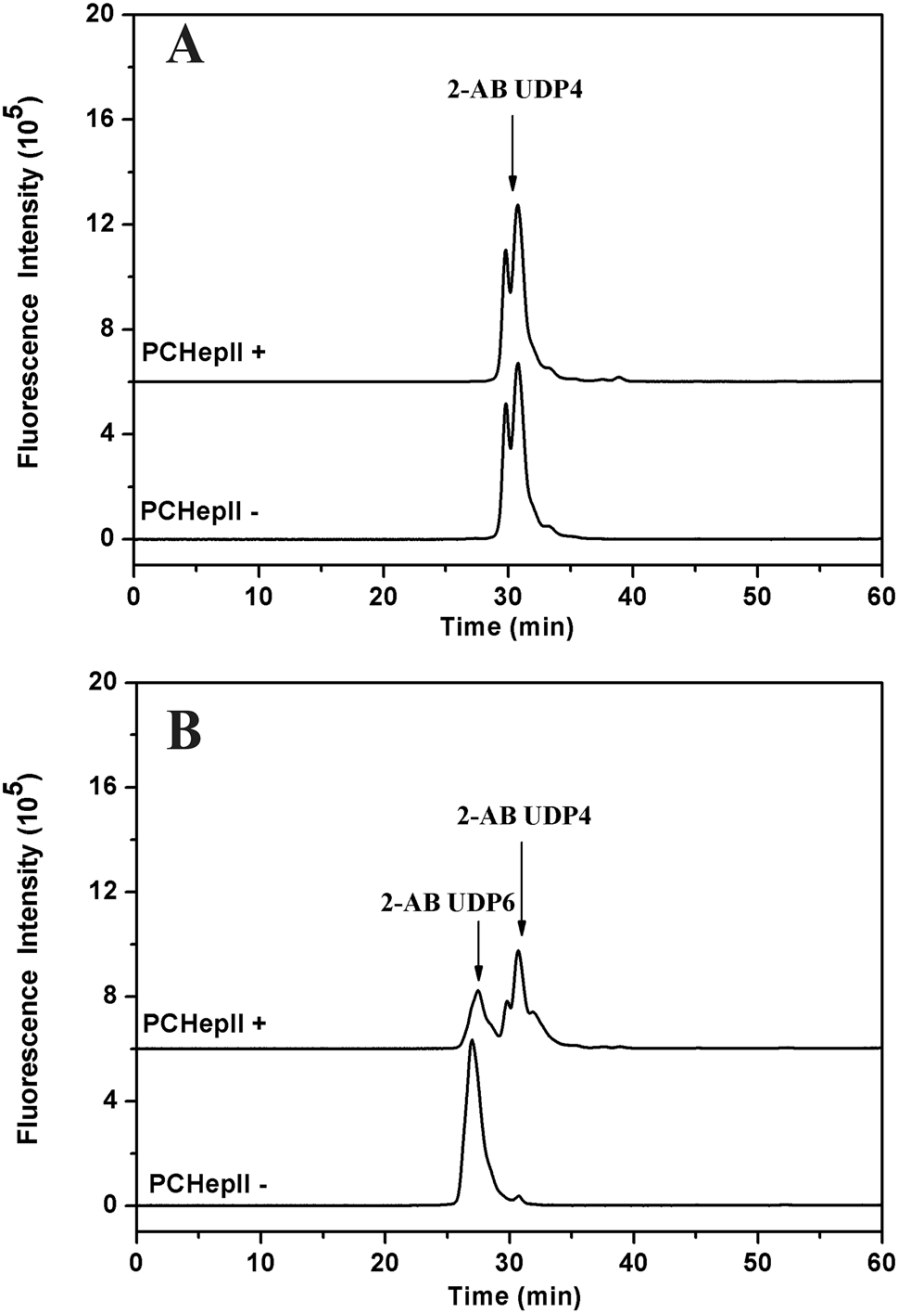
2-AB labeled oligosaccharides digestion analysis of PCHepII. 2 μg of the HP UDP4 (**A**) and UDP6 (**B**) were 2-AB labeled and then treated with excessive PCHepII for overnight. The samples were filtered and analyzed gel filtration HPLC with a fluorescence detector.

Fluorescence labelling is commonly used in the structural analysis of GAGs^34^. However, it has been observed that the fluorophores located at the end of the GAG chains often affect the degradation processes carried out by lyases^35^. To investigate the impact of fluorescence labelling on the digestion capacity of PCHepII, the HP oligosaccharides UDP4 and UDP6 were labelled with 2-AB (2-aminobenzamide) and then treated with PCHepII. The results showed that PCHepII could digest 2-AB-UDP6 to form 2-AB-UDP4 (Fig. 7B), but it was unable to generate 2-AB-UDP2 from 2-AB-UDP4 (Fig. 7A). While, PCHepII could efficiently degrade the unlabeled UDP4 (Supplementary Fig. 4A) and UDP6 (Supplementary Fig. 4B) to generate the disaccharide products. These findings suggested that PCHepII could not degrade the 2-AB-labelled tetrasaccharides, possibly because the fluorophores at the reducing end hampered the interaction of 2-AB-UDP4 with the active centre of PCHepII.

### Substrate size preference of PCHepII

**Figure 8:**
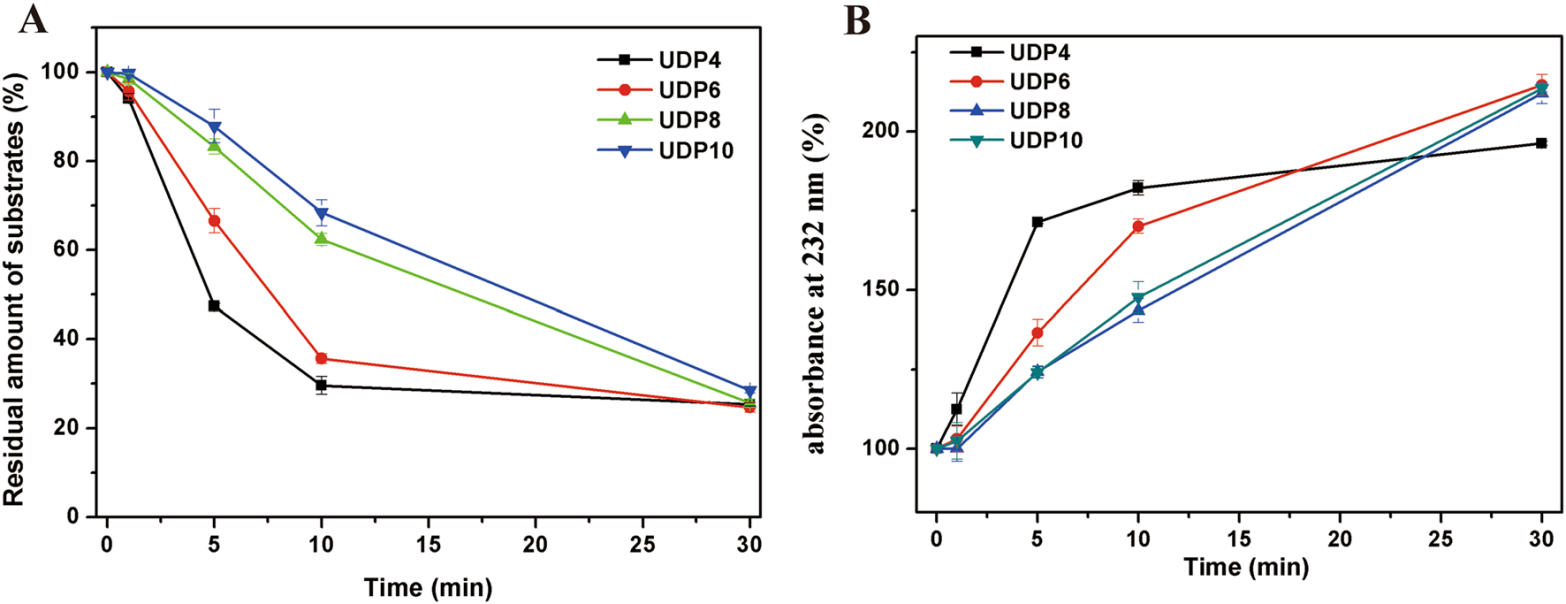
Size defined substrates degradation analysis by PCHepII. Each 2 nmol of the size defined substrates including UDP4, UDP6, UDP8 and UDP10 were treated with PCHepII for 0 min, 1 min, 5 min, 10 min, and 30 min. And then the samples were analyzed by UV gel-filtration HPLC and calculate the production of the unsaturated products (**A**) and residual amount of the substrates (**B**). Each data was shown as the mean of three replicates ± standard deviation.

To determine the substrate preference of PCHepII, unsaturated HP oligosaccharides of various sizes (UDP4, UDP6, UDP8, and UDP10) were employed and treated with PCHepII for time intervals ranging from 0 to 30 min. Figure 8A and B show changes in the unsaturated products and the monitored substrates, respectively. As time progressed, the amounts of unsaturated products increased and exhibited different trends (Fig. 8A). The production rate of unsaturated products formed from the degradation of UDP4 by PCHepII is higher than that of other oligosaccharides, especially at 5 min (Fig. 8A). Additionally, Fig. 8B demonstrates that the degradation rate of UDP4 was significantly higher than that of other oligosaccharides throughout the reaction process. These findings suggested that PCHepII prefers to catalyse smaller substrates, which explains why PCHepII generates small amounts of larger oligosaccharides when digesting HP polysaccharides in the early degradation stage (Fig. 6). This is because PCHepII primarily catalyses small-sized substrates after disrupting the polysaccharides, resulting in relatively lower enzyme activity towards HP polysaccharides.

### Mutagenesis study of PCHepII

**Figure 9:**
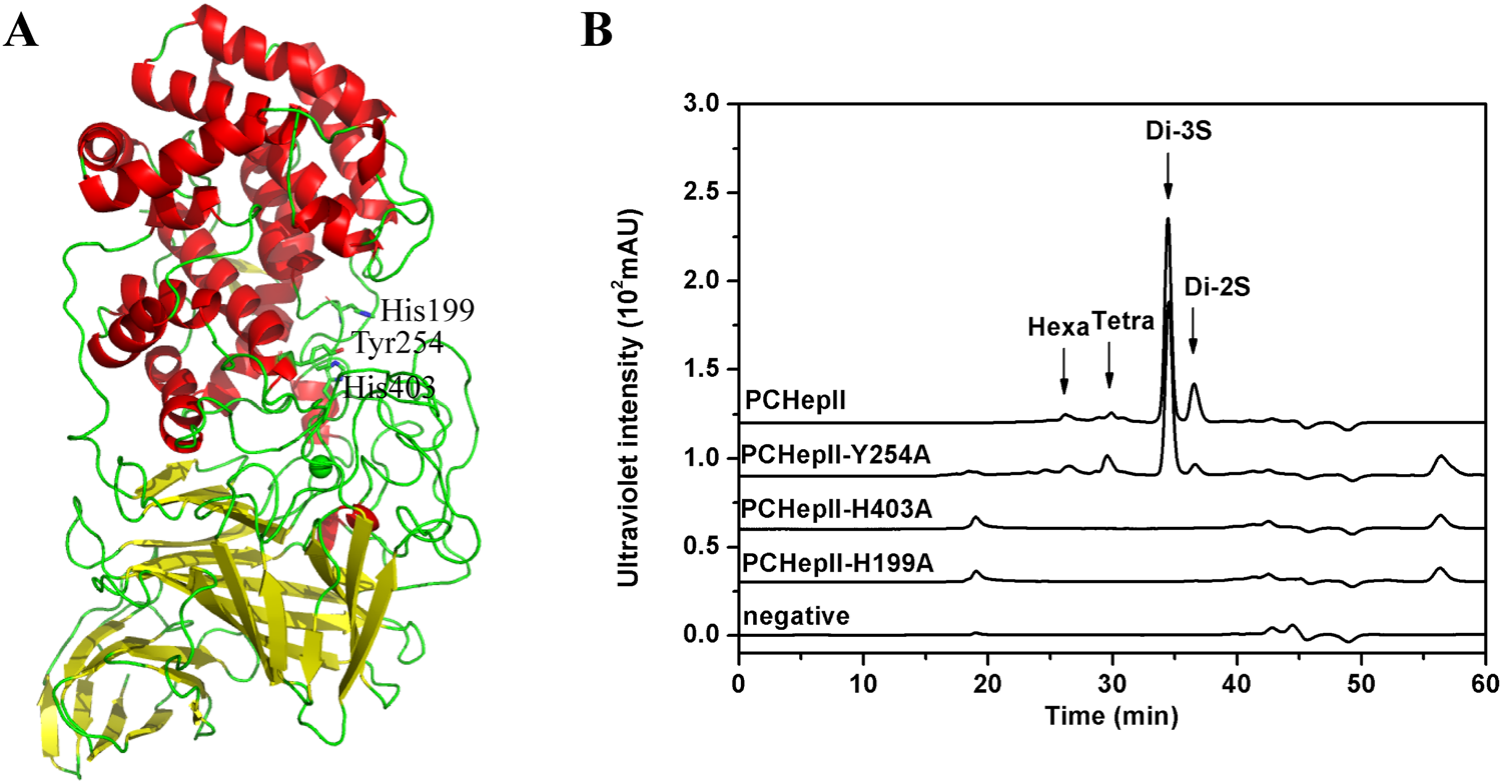
Simulated structure (**A**) and mutagenesis analysis (**B**) of PCHepII. (**A**) The structure of PCHepII was simulated via the Swiss Model online software. (**B**) The mutants PCHepII-H199A, PCHepII-Y254A, and PCHepII-H403A were applied to treat HP for overnight and then analyzed by the gel filtration HPLC. Hexa, HP unsaturated hexasaccharide; Tetra, HP unsaturated tetrasaccharide; Di-3S, the trisulfated HP unsaturated disaccharides; Di-2S, the disulfated HP unsaturated disaccharides.

To investigate the catalytic mechanism of the enzyme, the simulated structure of PCHepII was established using Swiss Model online software. Candidate active sites, including His^199^, Tyr^254^, and His^403^ (Fig. 9A), consistent with the active sites His^202^, Tyr^257^, and His^406^ of PHHepII^15^, were selected and then mutated to Ala individually. The mutants PCHepII-H199A and PCHepII-H403A completely lost enzyme activity towards the HP substrate (Fig. 9B), and the mutant PCHepII-Y254A retained the ability to catalyse HP (Fig. 9B), while the exact enzyme activity of PCHepII-Y254A was too low to be exactly calculated. These results indicated that the His^199^, Tyr^254^, and His^403^ residues play crucial roles in the catalytic action of PCHepII and may serve as the active centre.

## Discussion

Hepases are a large class of polysaccharide lyases that play a crucial role in HP and HS structural studies and in the preparation of LMWHs^1^. In recent decades, several hepases have been reported and can be classified into three categories: hepase I, hepase II, and hepase III^29^. However, these identified hepases were all derived from terrestrial bacteria, and hepases derived from marine bacteria are still unknown^30^. Due to significant differences in environment, hepases derived from marine bacteria may exhibit unique characteristics and have the potential to generate new products when applied in the production of HP oligosaccharides. In this study, a novel hepase named PCHepII, which was derived from the marine bacterium *Puteibacter caeruleilacunae,* was studied in detail.

Phylogenetic analysis revealed that PCHepII clusters with the Hepase II clades and shares the highest similarity of 68.07% with PHHepII^15^. Therefore, PCHepII can be considered a new member of the Hepase II family. However, it was surprising that PCHepII showed a preference for catalysing HP substrates rather than HS substrates, as it did not effectively degrade HS. Furthermore, analysis of the final HP and HS degradation products with PCHepII showed that the enzyme mainly generates highly sulfated disaccharides, including the trisulfated disaccharide ΔUA2S1-4GlcNS6S and the disulfated disaccharides ΔUA2S1-4GlcNS and ΔUA1-4GlcNS6S. While the final products of HP and HS, especially HS, consist of trisulfated and disulfated disaccharides, a large amount of the monosulfated disaccharides ΔUA1-4GlcNS and ΔUA1-4GlcNAc6S and the nonsulfated disaccharide ΔUA1-4GlcNAc were also observed. These characteristics clearly showed that PCHepII preferred to catalyse the highly sulfated regions of HP and HS, and the characteristics were different from those of the identified hepase II enzymes, which do not exhibit such substrate selectivity between HP and HS substrates^36,37^. Instead, the preference of PCHepII for degrading highly sulfated HP polysaccharides aligns more closely with hepase I^36,37^. This suggested that PCHepII could be classified as a novel type of Hepase, despite its high similarity to the hepase II family.

PCHepII showed higher activity at neutral pH and temperatures of 20°C and 30°C, similar to other GAG lyases found in marine bacteria^30^, consistent with marine environmental conditions. The optimal temperature for PCHepII was completely different from that of PHHepII (optimal temperature of 40°C) and other identified HepaseII^21^. PCHepII remained stable at 0–30°C even after 24 h and retained 78% of its enzyme activity. However, its stability drastically decreased at temperatures above 40°C, suggesting that PCHepII is a hypothermophilous enzyme. Additionally, PCHepII was more activated in a high salt environment, and the enzyme activity of PCHepII was enhanced with 100 mM KCl or NaCl. Even when the concentration of KCl or NaCl reached 250 mM, the enzyme activity of PCHepII was enhanced (KCl) or was not significantly affected (NaCl). This phenomenon was not observed in PHHepII, which was inhibited by salt^21^. These characteristics indicated that the marine-derived hepase PCHepII was different from terrestrial hepases and was more adapted to a marine environment.

The activity of PCHepII was significantly enhanced by the presence of 5 mM Ba^2+^ (145%), while it was completely inhibited by EDTA, indicating the essential role of divalent cations in its activity. However, for PHHepII, none of the divalent cations could enhance the activity of PHHepII^21^. Other heavy metal ions, including Ni^2+^, Co^2+^, Zn^2+^, Cu^2+^, Fe^2+^, Fe^3+^, Cr^3+^, Ca^2+^, Ag^+^, Pb^2+^, Hg^2+^, and Cd^2+^, could inhibit the enzyme activity of PCHepII, similar to PHHepII^21^. These results indicate that heavy metal ions may have impeded the reaction of hepases with substrates. The enzyme activity of PCHepII towards HP was calculated to be 254 mU/mg protein, and the exact enzyme activity towards HS was too low to be measured, although PCHepII could slowly degrade HS. The enzyme activities of PCHepII towards the substrates were far less than those of PHHepII, for which the enzyme activities towards HP and HS were 19 U/mg protein and 36.5 U/mg protein^38^, respectively. The results indicated that the enzyme activity of marine-derived hepases may be lower than that of terrestrial hepases, and this difference should be further analysed via the identification of more marine-derived hepases.

Similar to Hepase II, PCHepII exhibited an endolyase form when degrading substrates^1^. However, it is interesting to note that PCHepII primarily generates disaccharide products during the catalytic stages, with a relatively low amount of larger oligosaccharides. Typically, endolyases randomly digest polysaccharide chains, resulting in a higher production of larger oligosaccharides in the early stages of digestion, which are then converted to disaccharides^39^. Further testing revealed that PCHepII showed a preference for catalysing small-sized substrates, particularly HP tetrasaccharides. The results indicated that when PCHepII degrades substrates, it may randomly combine with the chains and then combine with the generated unsaturated oligosaccharides and sequentially catalyse the release of disaccharides from the oligosaccharides, which explains why disaccharide peaks dominate the products throughout the digestion stages. Consequently, the enzyme activity of PCHepII towards HP is relatively low.

Fluorescence labelling is a common and straightforward method used in structural studies of GAGs^34^. Lyases that can breakdown 2-AB labelled disaccharides from oligosaccharides are valuable tools and have already been utilized in the structural studies of chondroitin sulfate^35^. However, for HP/HS, studies of this type of enzyme were relatively scarce. PCHepII was able to breakdown 2-AB-labelled hexasaccharides and generated 2-AB-labelled tetrasaccharides, but it was inactive towards 2-AB-labelled tetrasaccharides. This indicated that the fluorophore 2-AB at the reducing end of the oligosaccharides may impede necessary interactions between the substrates and the active centre of PCHepII and thus reduce the degradation capacity of PCHepII.

The His^199^, Tyr^254^, and His^403^ residues were selected as candidate active centre residues through alignment with PHHepII^40^. The mutants PCHepII-H199A, PCHepII-Y254A, and PCHepII-H403A were tested for residual enzyme activity using HP polysaccharides as substrates. The enzyme activities of the mutants were either completely absent or greatly reduced. Therefore, it can be concluded that the three residues His^199^, Tyr^254^, and His^403^ form the active centres of PCHepII and are essential for stabilizing and facilitating proton transfer in HP/HS substrates.

## Conclusions

In this study, a marine bacteria-derived hepase, PCHepII, was studied in detail, and its characteristics were examined. PCHepII demonstrated excellent solubility and expression levels, making it suitable for industrial production. Additionally, PCHepII exhibited preferential activity in environments with intermediate to low temperatures and high salt concentrations, which are conditions commonly found in marine environments. Despite having endo-lyase activity, PCHepII displayed higher activity towards small substrates. This is the first study to report the marine-derived hepase, PCHepII, and the results might provide a potential approach for HP and HS research in the future.

### Supplementary Information

Supplementary Figures.

## Data Availability

All data generated or analysed during this study are included in this published article. The original gel figure of PCHepII was shown in supplementary data.
