# Supplementary material for: Identification and characterization of a novel heparinase PCHepII from marine bacterium Puteibacter caeruleilacunae
Source: Sci Rep. 2023 Nov 17;13:20112. doi: 10.1038/s41598-023-47493-y (PMC10656541; doi:10.1038/s41598-023-47493-y)
Supplement: Supplementary file 1 — Supplementary Figures. [file 41598_2023_47493_MOESM1_ESM.pdf]

Supplementary materials for this manuscript include the following:

Supplementary Information for

Identification and characterization of a novel heparinase PCHepII from marine bacterium *Puteibacter caeruleilacunae*

Danrong Lu, Luping Wang, Zeting Ning, Zuhui Li, Meihua Li, Yan Jia and Qingdong Zhang

Corresponding authors: zhangqingdong@wfmc.edu.cn

Supplementary Information Text

Supplementary Methods

Preparation of the unsaturated HP oligosaccharides.

To obtain the unsaturated HP oligosaccharides prepared by the degradation of HP by Hepase I, the reaction system containing 100 μL enzyme (3 mU/μL), 100 μL HP (10 mg/mL), 333 μL sodium phosphate buffer (150 mM, pH 8.0) and 533 μL deionized water were applied. The mixture was then incubated at 37°C for 30 min and then the reaction mixture was boiled for 10 min. The samples were centrifuged at 15000×g for 10 min and then filtered through 0.22 μm filters followed by analyzed using gel filtration HPLC on Superdex 30 Increase 10/300 GL column (Cytiva). The elution process was performed using 0.2 M NH_4HCO_3 at a flow rate of 0.4 mL/min, the fractions were monitored at 232 nm sing a UV detector. The different sized HP oligosaccharides include UDP4, UDP6, UDP8, and UDP10 were collected and freeze-dried repeatedly to remove NH_4HCO_3 to get the purified oilosaccharides.

Supplementary Figures

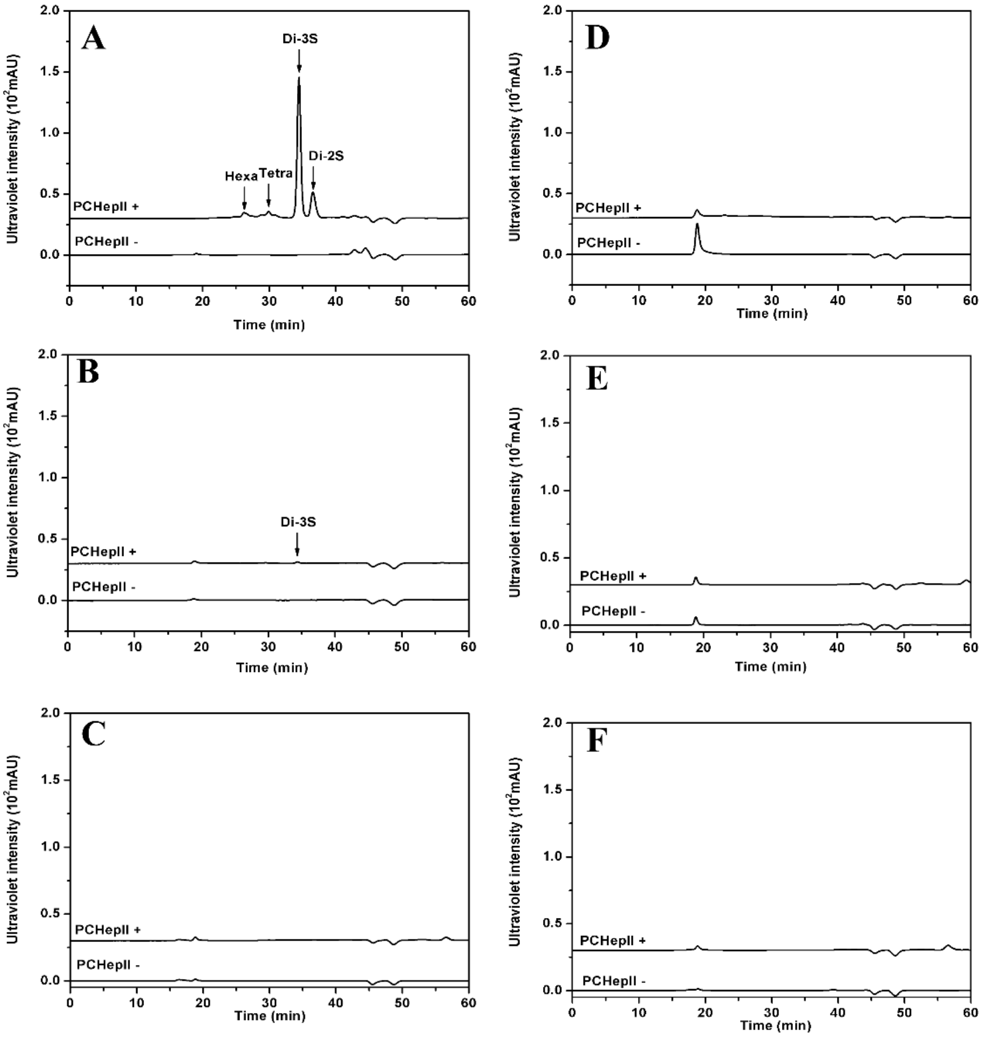

Supplementary Figure 1 Substrate specificity analysis of PCHepII. Substrates HP (A), HS (B), HA (C), CS (D), DS (E), and alginate (F) were treated with PCHepII and analyzed via the gel filtration HPLC. Di-3S, the trisulfated HP and HS unsaturated disaccharides; Di-2S, the disulfated HP and HS unsaturated disaccharides; Tetra, HP and HS unsaturated tetrasaccharide; Hexa, HP and HS unsaturated hexasaccharide.

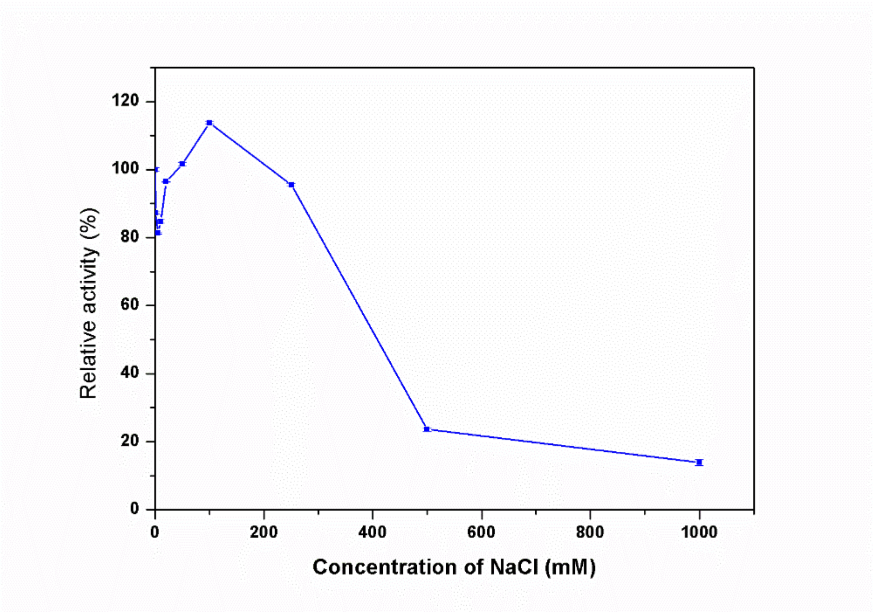

Supplementary Figure 2 Effect of NaCl concentration on PCHepII activity. The activity of PCHepII without NaCl was set as 100%.

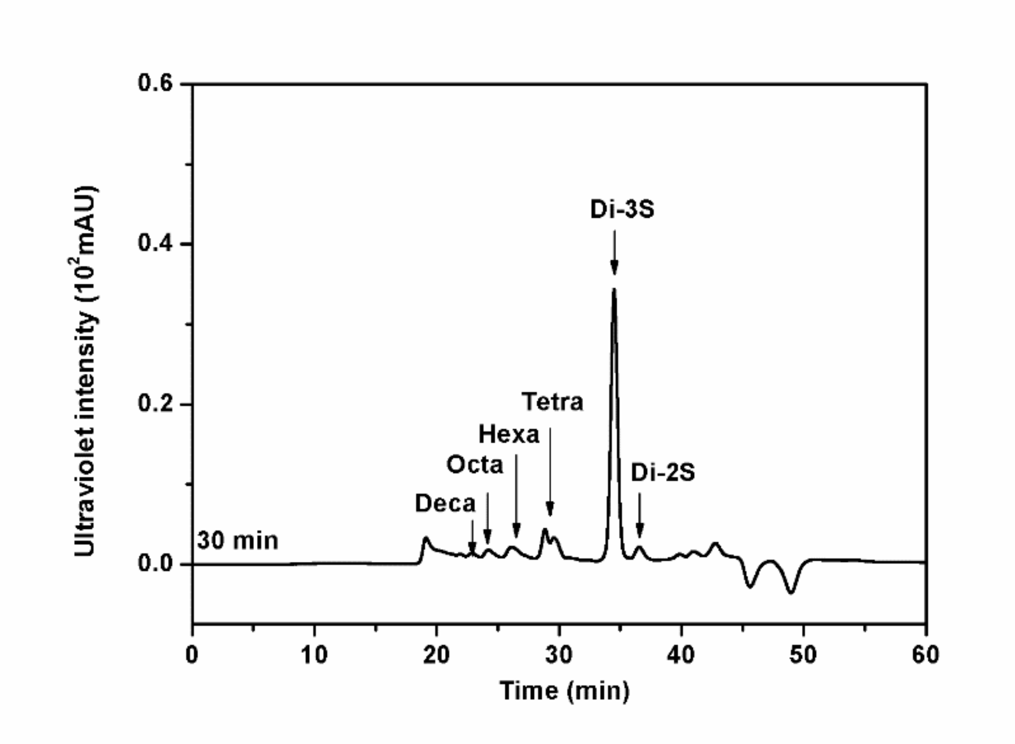

Supplementary Figure 3 The degradation of HP by PCHepII at 30 min. The degree of depolymerization of HP unsaturated oligosaccharides released from the polysaccharide substrates by the digestion of HP are indicated by arrows: Deca, HP unsaturated decasaccharide; Octa, HP unsaturated octasaccharide; Hexa, HP unsaturated hexasaccharide; Tetra, HP unsaturated tetrasaccharide; Di-3S, the trisulfated HP unsaturated disaccharides; Di-2S, the

disulfated HP unsaturated disaccharides.

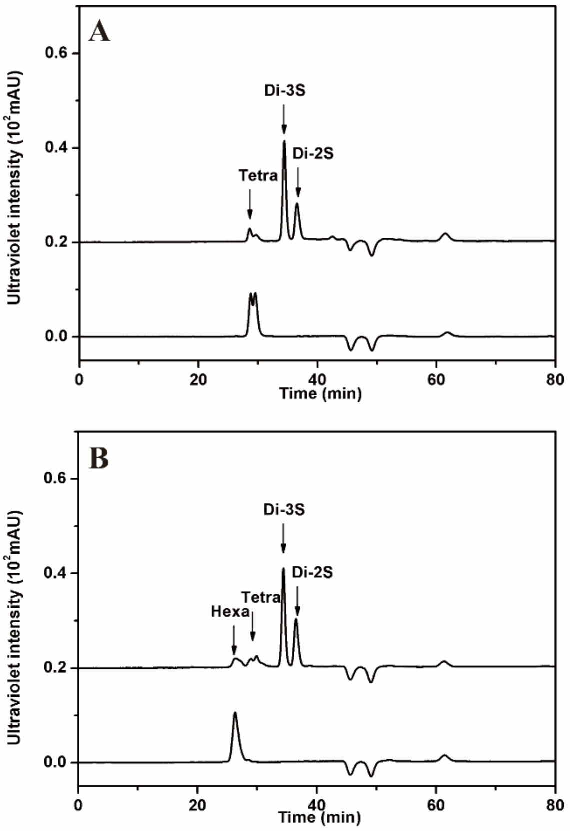

Supplementary Figure 4 The degradation of HP tetrasaccharides and hexasaccharides by PCHepII. The degree of substrates released from the substrates by the digestion of HP are indicated by arrows: Hexa, HP unsaturated hexasaccharide; Tetra, HP unsaturated tetrasaccharide; Di-3S, the trisulfated HP unsaturated disaccharides; Di-2S, the disulfated HP unsaturated disaccharides.

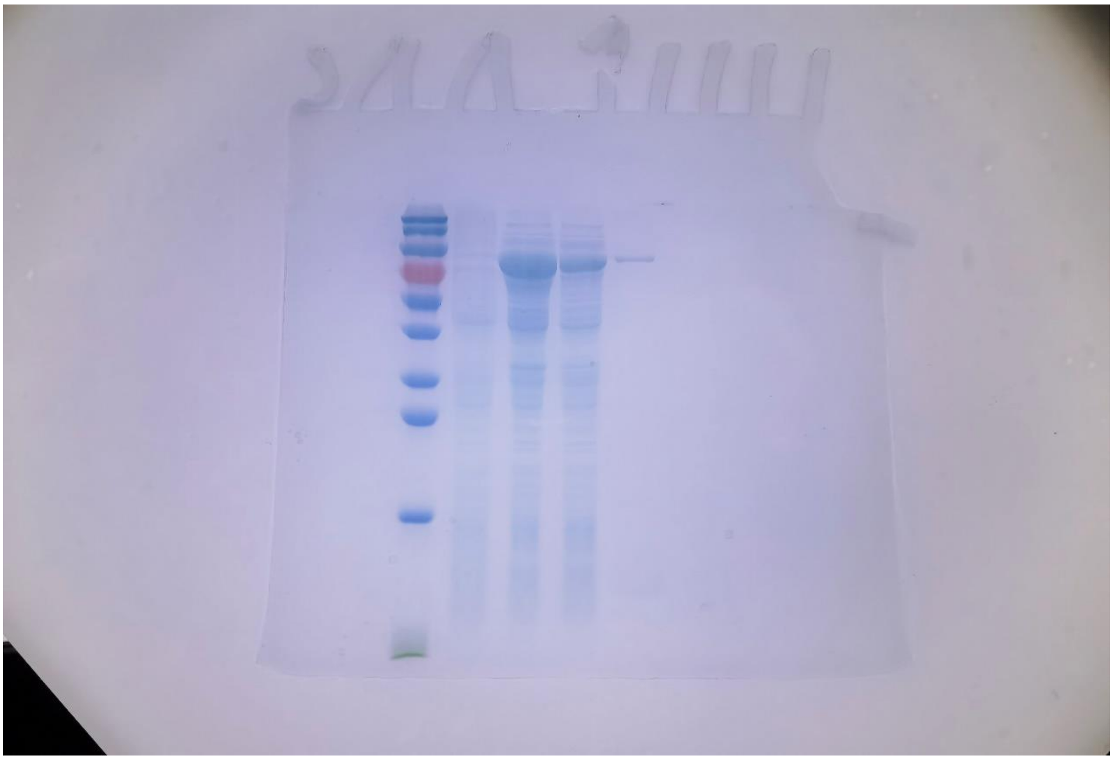

1 2 3 4 5

Supplementary Figure 5 The original gel figure of PCHepII. 1, pre-stained protein standard marker (15-180 kDa); Lane 2, uninduced cell lysate of *E. coli*-pET30a; Lane 3, IPTG-induced cell lysate of *E. coli*-pET30a-*PCHepII*; Lane 4, the induced lysate supernatant of *E. coli*-pET30a-*PCHepII*; Lane 5, purified PCHepII after Ni^{2+} affinity chromatography.
